# Advancing Exposure Science through Chemical Data Curation and Integration in the Comparative Toxicogenomics Database

**DOI:** 10.1289/EHP174

**Published:** 2016-05-12

**Authors:** Cynthia J. Grondin, Allan Peter Davis, Thomas C. Wiegers, Benjamin L. King, Jolene A. Wiegers, David M. Reif, Jane A. Hoppin, Carolyn J. Mattingly

**Affiliations:** 1 Department of Biological Sciences, North Carolina State University, Raleigh, North Carolina, USA; 2 Department of Bioinformatics, The Mount Desert Island Biological Laboratory, Salisbury Cove, Maine, USA; 3 Center for Human Health and the Environment, North Carolina State University, Raleigh, North Carolina, USA

## Abstract

**Background:**

Exposure science studies the interactions and outcomes between environmental stressors and human or ecological receptors. To augment its role in understanding human health and the exposome, we aimed to centralize and integrate exposure science data into the broader biological framework of the Comparative Toxicogenomics Database (CTD), a public resource that promotes understanding of environmental chemicals and their effects on human health.

**Objectives:**

We integrated exposure data within the CTD to provide a centralized, freely available resource that facilitates identification of connections between real-world exposures, chemicals, genes/proteins, diseases, biological processes, and molecular pathways.

**Methods:**

We developed a manual curation paradigm that captures exposure data from the scientific literature using controlled vocabularies and free text within the context of four primary exposure concepts: stressor, receptor, exposure event, and exposure outcome. Using data from the Agricultural Health Study, we have illustrated the benefits of both centralization and integration of exposure information with CTD core data.

**Results:**

We have described our curation process, demonstrated how exposure data can be accessed and analyzed in the CTD, and shown how this integration provides a broad biological context for exposure data to promote mechanistic understanding of environmental influences on human health.

**Conclusions:**

Curation and integration of exposure data within the CTD provides researchers with new opportunities to correlate exposures with human health outcomes, to identify underlying potential molecular mechanisms, and to improve understanding about the exposome.

**Citation:**

Grondin CJ, Davis AP, Wiegers TC, King BL, Wiegers JA, Reif DM, Hoppin JA, Mattingly CJ. 2016. Advancing exposure science through chemical data curation and integration in the Comparative Toxicogenomics Database. Environ Health Perspect 124:1592–1599; http://dx.doi.org/10.1289/EHP174

## Introduction

Exposure science plays a critical role in the translation and assessment of experimental toxicity data, prioritizing research, aiding risk analysis for human health, and informing public health decisions (Hubal 2009). Studying environmental exposures also facilitates characterization of the exposome, which is defined as the totality of an individual’s environmental exposures from the prenatal period onward (Wild 2005). The exposome complements the human genome by providing a measure of environmental exposure history in the three broad domains of internal, specific external, and general external exposures (Wild 2012). Further, it contributes to the expanded vision of exposure science proposed by the National Research Council (NRC), which incorporates internal and external exposure markers and extends exposure science both inward and outward from the contact point between a stressor and receptor. (Lioy and Smith 2013; NRC 2012).

Recent advances in exposure research include *a*) dedicated funding mechanisms and research programs, largely through the National Institute of Environmental Health Sciences (e.g., Exposure Biology Program and Children’s Health Exposure Analysis Resource); *b*) databases that are beginning to include exposure concepts [e.g., the Toxin and Toxin-Target Database (Wishart et al. 2015) which displays manually curated expression data from the Comparative Toxicogenomics Database (CTD; http://ctdbase.org/) in association with toxicants]; and *c*) large-scale analyses that incorporate exposome measurements in population-based studies [e.g., Environment-Wide Association Studies (Patel and Ioannidis 2014) and Human Phenotype Network (Darabos et al. 2014)]. Despite these advances, exposome-dedicated research has lagged behind genomic studies. Our work provides a new exposome research resource by providing curated data from the published literature on chemical stressors and their interactions with humans (i.e., “receptors”), centralizing and harmonizing the data, and integrating this information with CTD chemical–gene/protein–disease relationships.

Since 2005, we have been building and expanding the CTD to improve understanding of the effects of environmental exposures on molecular pathways and disease outcomes (Davis et al. 2015). CTD biocurators manually curate chemical–gene, chemical–disease and gene–disease relationships from the literature and integrate these associations to construct chemical–gene–disease networks. Currently, there are 27 million toxicogenomic relationships in the “core” CTD that connect chemicals, genes/proteins, diseases, phenotypes, molecular networks, Gene Ontology (GO) annotations, and pathways (Davis et al. 2009, 2011, 2015). These relationships can be explored with the CTD’s visualization and analysis tools to help elucidate molecular mechanisms underlying environmental diseases. The CTD is recognized as a vital resource to the research community, with > 60 databases linking to CTD data. In addition, the CTD has been cited in > 800 peer-reviewed articles, with CTD data being used in such diverse studies as identifying tumor risks in children of pesticide-exposed parents (Kunkle et al. 2014) and predictive toxicology modeling (Audouze and Grandjean 2011; Hu et al. 2015).

To support the scientific community’s need for centralization and integration of exposure data into a broader biological framework, we expanded core CTD content to include exposure data, specifically within the context of chemical stressors and human receptors. To capture these data, we used the Exposure Ontology (ExO), a framework that structures key exposure concepts: “exposure stressor” (an agent, stimulus, activity or event that causes stress on an organism), “exposure receptor” (an entity that interacts with an exposure stressor), “exposure event” (an interaction between an exposure stressor and an exposure receptor), and “exposure outcome” (an entity that results from an exposure event) (Mattingly et al. 2012). Here, we describe the initial phase of exposure curation and provide examples that illustrate the impact of CTD-mediated analyses of exposure data, including a meta-analysis of chemical–disease interactions from the Agricultural Health Study (AHS).

The AHS is a prospective study investigating the role of agricultural exposures in the development of cancer and other chronic illnesses in a cohort of ~90,000 pesticide applicators and their spouses in Iowa and North Carolina (Alavanja et al. 1996). In the initial phase of exposure data curation, we prioritized coverage of the AHS to use as a case study because it *a*) has generated numerous publications but has not yet undergone a meta-analysis; *b*) covers a broad range of chemicals and diseases with significant overlap in the CTD; and *c*) provided opportunities for informed and immediate feedback from a former AHS principal investigator, J. Hoppin, who collaborated with us on early development of ExO and on our curation process.

Development of an exposure module in the CTD complements other public toxicology resources and initiatives such as the U.S. Environmental Protection Agency’s (EPA’s) Aggregated Computational Toxicology Resource (Judson et al. 2008), which is a warehouse of chemical toxicity data, and the European Chemicals Agency Registration, Evaluation, Authorization and Restriction of Chemicals (Foth and Hayes 2008), which addresses the production and use of chemicals. Although these organizations play substantial roles in exposure research, the CTD provides a unique knowledge base of exposure data that incorporates chemical–gene–pathway–outcome information. Inclusion of exposure data in the CTD also responds directly to the need for informatics technologies to advance exposure science research as expressed by the NRC (2012).

## Methods

### Literature Triage for Exposure Study Curation

Following established curation practices at the CTD (Davis et al. 2009, 2011), peer-reviewed journal articles were prioritized for exposure curation by querying MEDLINE from PubMed (http://www.ncbi.nlm.nih.gov/pubmed/) (Vastag 2000) using a generic exposure-themed query: “environmental exposure” AND human NOT review AND hasabstract AND English (lang). Articles were filtered for publication within the last 10 years and free full-text availability to ensure user access to the source information. We acknowledge that this query is not all-inclusive, but it provided an initial corpus for evaluation; we anticipate an expanded and iterative querying and triaging process as we go forward. As with our core data, exposure curation is an ongoing process, with new information being loaded into CTD on a monthly basis (http://ctdbase.org/about/dataStatus.go).

### ExO Development and Implementation

To standardize exposure curation and facilitate integration and search capabilities in the CTD, the ExO framework was expanded to include terms describing a subset of the exposome specifically involved in chemical exposures and human health outcomes. ExO depth was expanded by using existing third-party vocabularies where applicable: MeSH (Coletti and Bleich 2001) for chemical and anatomical terms; MEDIC (Davis et al. 2012) for disease terms; Gene Ontology (GO) (Ashburner et al. 2000) for biological processes (“phenotypes”); and NCBI Gene (Maglott et al. 2011) for official gene symbols. For geographic location, country codes published by the International Organization for Standardization (ISO 3166; http://www.iso.org/iso/country_codes) and U.S. state abbreviations were used. To annotate ethnicity and race, terms from the PhenX toolkit were customized (Hamilton et al. 2011). Structured terms were also developed in coordination with curation to describe stressor sources, receptors, smoking status, influencing health factors, and correlations between stressors and diseases or phenotypes. As this project proceeds, “orphan” terms will be standardized and others mapped to existing ontologies so that ExO evolves, is nonredundant, and ensures consistent use among the research community.

### Exposure Curation

In accordance with the CTD’s specific objectives, exposure articles must report chemical stressors affecting human receptors. If an article described a nonchemical stressor or a nonhuman receptor, the article was not further reviewed. For articles meeting these criteria, CTD biocurators read the article and then composed “exposure statements,” which capture relevant data in specified fields within a single row of a Microsoft Excel spreadsheet. Each exposure statement records information about a stressor–receptor–event–outcome from an exposure study, defined by a unique PubMed Identifier. Numerous exposure statements are typically composed for each article to capture a high level of granularity and to accurately reflect the complexity of the information. For example, a study may report measurements of an exposure biomarker level for multiple chemicals, or measurements may be different for different subsets of a cohort. Curated data are validated through a multipoint, rule-based computational process that integrates third-party and CTD-specific vocabularies. This process confirmed compliance with our curation policies (see Table S1), ensured integration with data in the core CTD, and provided the foundation for implementing more complex search capabilities planned for future releases. Exposure data are loaded into the CTD’s PostgreSQL database management system and made available through the public web interface. Validation and load processes are primarily Java-based and run in a Linux environment.

### Database Engineering and Architecture

The CTD’s database architecture is well documented (Davis et al. 2011). Briefly, the CTD is composed of three major databases, including a third party database, which contains transient data extracted from external sources (e.g., MeSH); the curation database, which contains persistent data manually curated by CTD scientists; and the public web application (PWA) database, which integrates data from the curation and the third party databases. The PWA database is the sole data source for the CTD’s public web application and is designed as a high-speed reporting database. Exposure data are initially loaded to CTD’s curation database; then, the data are consolidated and integrated with data from the third party database and loaded to the PWA database. A total of 49 new tables, comprising 239 columns, were added to the curation and PWA databases for this data-intensive exposure module. These tables are grouped into five high-level categories: published article, stressor, receptor, event, and outcome.

### Agricultural Health Study (AHS) Data Analysis

At the time of analysis (July 2015), 99 of the 111 articles related to the AHS contained interactions between 62 chemical stressors and 46 disease outcomes, with the remaining 12 articles reporting biomarker measurements or phenotypic outcomes for 30 chemical stressors. Positive and negative associations reported as statistically significant in peer-reviewed articles were curated (definitions of significance varied in data sets, see Supplemental Material, “Part 1. Agricultural Health Study”). In addition, we captured the central “take-home” points emphasized by the authors; for example, results that were highlighted by authors but did not reach author-defined statistical significance were still curated but were coded as a predictive or hypothetical relationship. Curated chemical-disease interactions were assigned numerical values based on the number of positive, negative, or null interactions, as reported by the author and were represented as a matrix, see Supplemental Material, “Part 1. Agricultural Health Study.” The R/ComplexHeatmap package (version 1.0.0; https://github.com/jokergoo/ComplexHeatmap) was used to perform single-linkage clustering of diseases, with chemicals sorted in decreasing order of overall interaction score.

CTD tools *Set Analyzer* (http://ctdbase.org/tools/analyzer.go) and *MyVenn* (http://ctdbase.org/tools/myVenn.go) were used to demonstrate how integration of exposure data with the CTD can add biological context to exposure information, see Supplemental Material, “Part 2. CTD Set Analyzer tool.” To gain perspective on how exposure and core CTD data complement each other, the number and type of disease outcomes for 18 AHS pesticides were compared based on their curated content from our exposure versus core data sets, see Supplemental Material, “Part 3. CTD MyVenn tool.” Overlapping and unique diseases between the exposure and core datasets were detected. Novel diseases provided from the core CTD were classified using MEDIC-Slim disease categories (Davis et al. 2012).

### Data Version

Analyses were based on data from our August 2015 monthly release (CTD revision 14263).

## Results

### CTD Exposure Curation Paradigm

**Figure 1:**
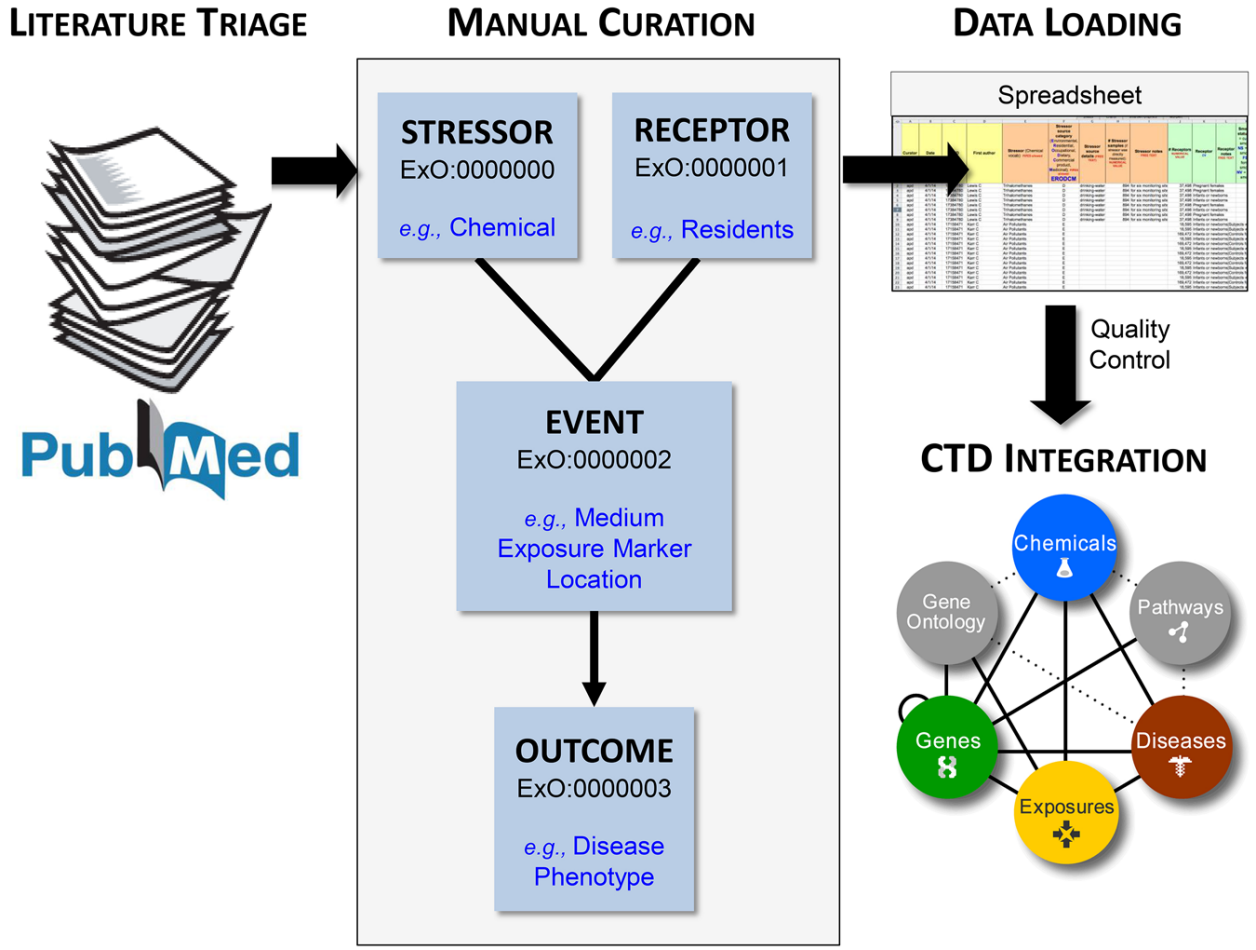
Comparative Toxicogenomics Database (CTD) exposure curation paradigm. Articles are retrieved and triaged from PubMed using a specialized query designed for environmental exposure science. The corpus is then manually curated by professional biocurators, anchoring the information to the four Exposure Ontology (ExO) concepts. Data are captured on spreadsheets and uploaded to the public CTD. Terms curated from exposure articles are directly integrated with the public CTD, including stressors integrated with CTD chemicals, markers integrated with CTD chemicals or genes, and outcomes integrated with CTD diseases and GO biological processes (“phenotypes”).

**Figure 2:**
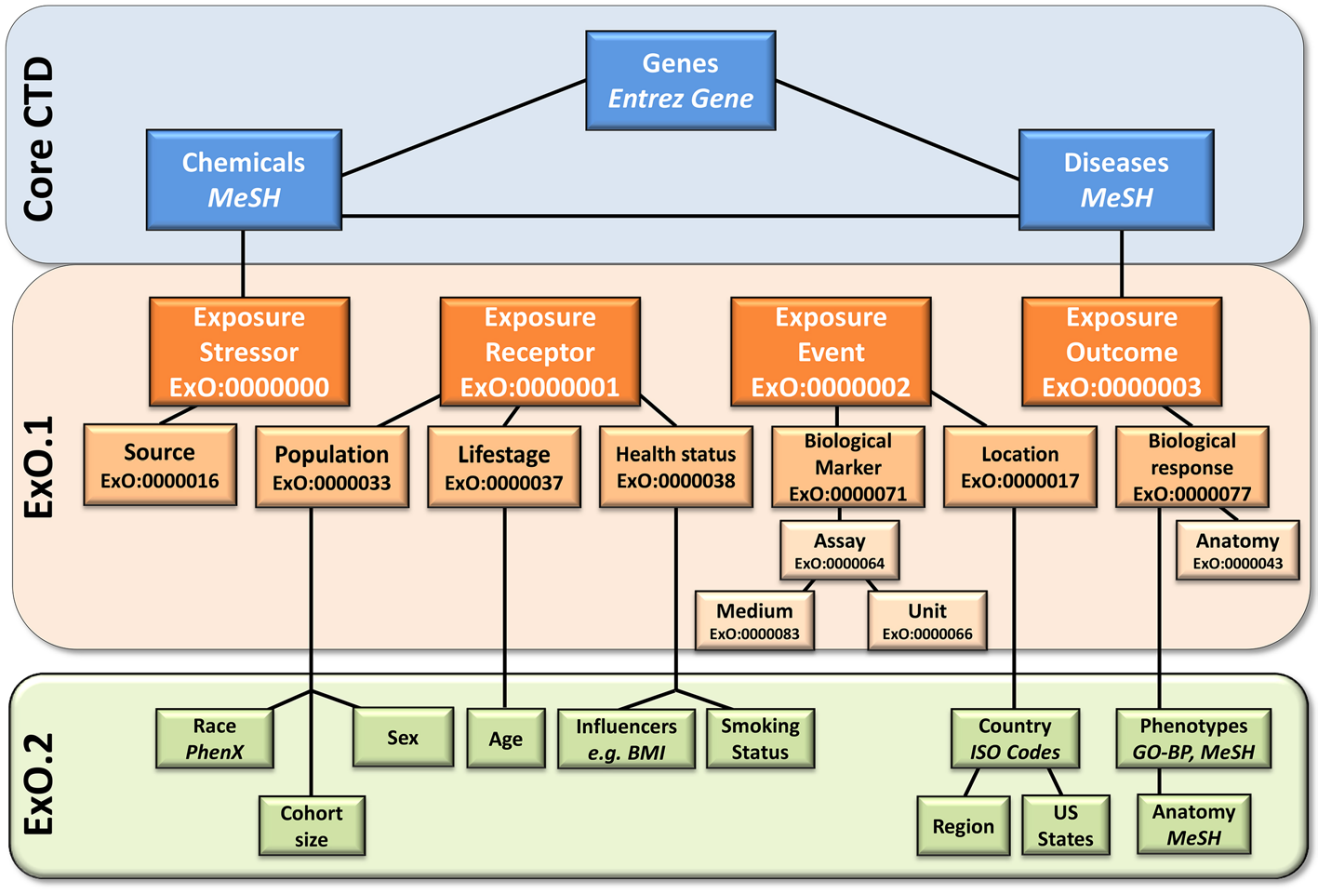
Schematic diagram of relationships among core Comparative Toxicogenomics Database (CTD), Exposure Ontology (ExO) concepts, and content being curated for the CTD. The central ExO terms “Exposure Stressor” and “Exposure Outcome” align directly with chemical and disease categories that are part of the core CTD (terms in blue). Terms in orange (ExO.1) have been previously described as part of the ontology. Terms in green (ExO.2) represent new components that have been expanded in coordination with curation of the CTD’s exposure module. BMI, body mass index; GO-BP, Gene Ontology–Biological Process; MeSH, Medical Subject headings.

The CTD exposure data curation paradigm (Figure 1) was designed through an iterative process with input from exposure scientists, several of whom helped develop ExO (Mattingly et al. 2012). To maximize information extracted from exposure articles and to ensure alignment with emerging interests in the research community, key concepts of the exposome were incorporated into the ExO structure. These concepts were further expanded in a data-driven manner in coordination with curation (Figure 2). The “Exposure Receptor” concept was expanded to include six new data categories to capture details of race/ethnicity, sex, cohort size, age, smoking status, and other factors influencing health status. “Exposure Event” was expanded to capture geographic location, including country, U.S. state, and region (e.g., city). These concepts are not the only ones being curated for the CTD; they are concepts that were added to the original ExO framework to accommodate the exposure curation goals for the CTD. We anticipate that ExO will continue to evolve as new data emerge, as CTD curation continues, and as the community articulates the need for additional expansion into different areas of the exposome (e.g., ecosphere).

Based on this expanded ExO framework and on ongoing iterations of test curation, a Microsoft Excel-based curation spreadsheet was created; this spreadsheet consisted of 54 data columns that capture 35 types of information pertaining to the five exposure categories: publication article, stressor, receptor, event, and outcome. To date, 1,712 triaged articles were reviewed, of which 1,067 (62%) were curated and 645 were rejected for lack of prioritized content for the CTD (e.g., our policy requires that the exposure stressor be a chemical). Approximately 53,000 curated exposure statements comprise information for 609 chemical stressors, 245 diseases, and 146 nondisease phenotypes, studied in 98 different countries (see Table S1).

### Data Access

**Figure 3:**
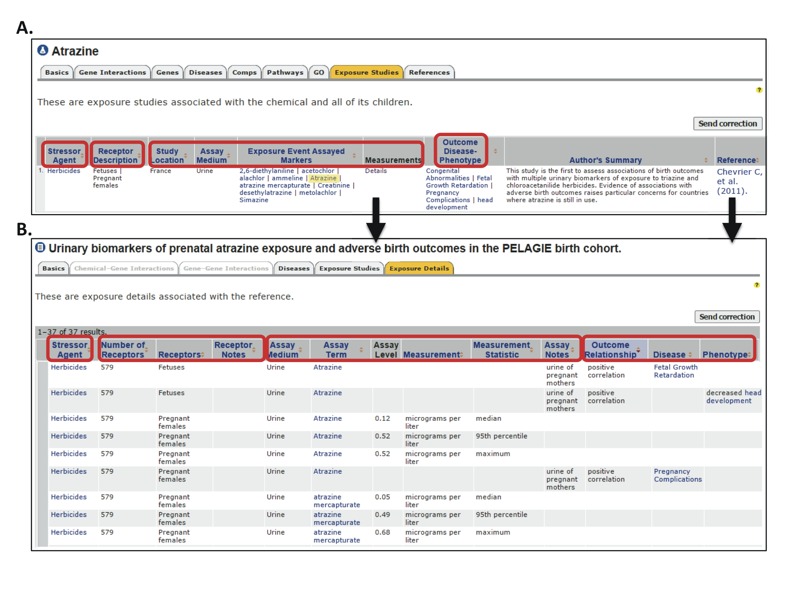
Exposure data are displayed in summary form on chemical, gene, disease, and Gene Ontology (GO) pages in the Comparative Toxicogenomics Database (CTD). (*A*) Exposure Ontology (ExO) concepts are represented by blue column headers (circled in red), including “Stressor Agent,” “Receptor Description,” “Exposure Event Study Location,” “Assay Medium and Assayed Markers,” and “Outcome Disease-Phenotype.” The stressor (CTD chemical term) is highlighted in yellow, with related terms in the chemical hierarchy. All mentioned chemical, gene, disease, and GO (“phenotype”) terms hyperlink to their individual CTD pages with additional details such as inference networks and gene and pathway enrichment analyses for disease terms. The “Author’s Summary” highlights the paper’s findings and provides context for the results. References link to exposure study details (*B*), including description and number of receptors, assay medium, specific levels of assayed markers, and the outcome relationship.

The CTD provides several options for accessing curated exposure data. A new “Exposure Studies” tab was added to our chemical, gene, disease, and GO pages. This tab provides a summary view of all curated exposure studies associated with the entity of interest (e.g., chemical), including receptor description, study location, assay medium, event markers, outcome, and a link to the primary reference (Figure 3A). Columns can be sorted by clicking headers, and results can be downloaded in various formats for further analysis. The “Author’s Summary” column highlights the article’s primary findings and provides context for the results. Exposure marker levels, receptor descriptions, and stressor–outcome interactions are found via the “Details*”* link under the “Measurements” column or on the reference page “Exposure Details” tab (Figure 3B). All chemical, gene, disease, and GO terms are hyperlinked to their respective CTD pages, providing seamless integration of exposure data with core CTD content.

### AHS Data

**Figure 4:**
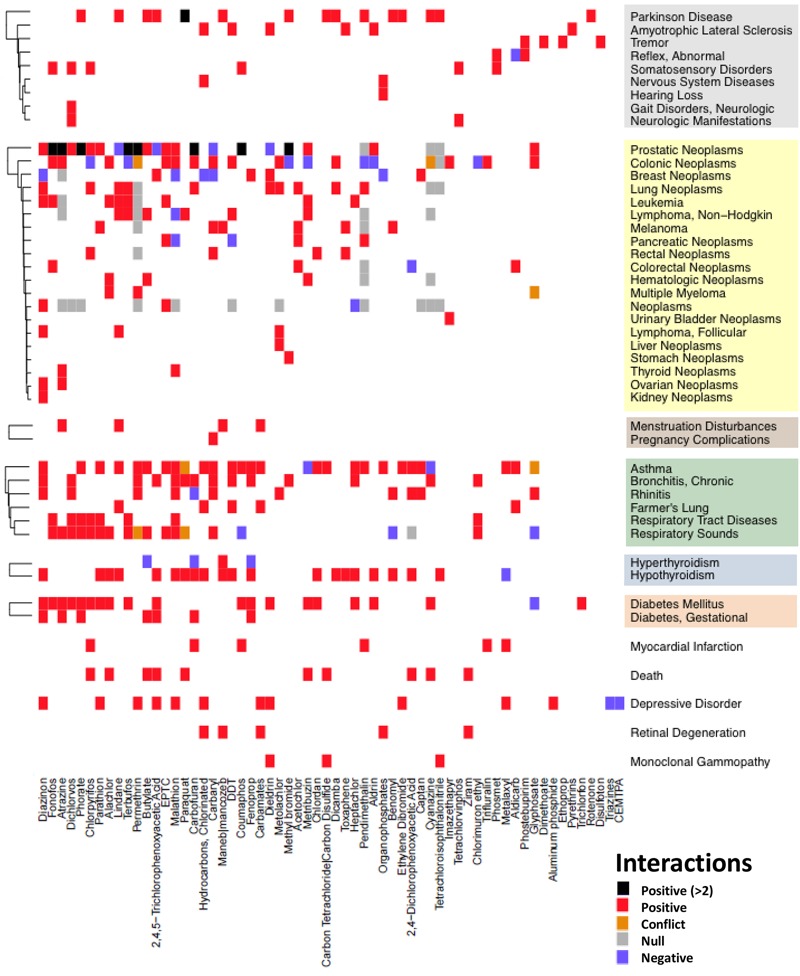
High-level view of reported chemical-disease interactions from Agricultural Health Study (AHS) articles. A heat map showing the relationships between 62 chemical stressors and 46 disease outcomes curated from 99 AHS articles. Chemical–disease correlations are coded as black (more than 2 significant or highlighted positive associations), red (1–2 positive associations), purple (significant negative associations), orange (conflicts between positive and negative), gray (null), and white (inconclusive, unreported, or unstudied). On the right-hand side of the figure, outcomes are clustered by their disease category: gray field (neurological diseases), yellow (cancer), tan (reproductive tract diseases), green (respiratory tract diseases), blue (endocrine system diseases), and orange (metabolic diseases). CEMTPA, 2-chloro-N-(ethoxymethyl)-N-[2-methyl-6-(trifluoromethyl)phenyl]acetamide; DDT, dichlorodiphenyltrichloroethane; EPTC, *S*-ethyl dipropylthiocarbamate.

To demonstrate how centralization and integration of exposure data in the CTD can increase understanding of human exposures and related health outcomes, we prioritized the AHS articles as a case study. Curation of 111 AHS articles yielded 1,552 exposure statements describing relationships between 89 chemicals and 56 diseases. After excluding data related to general terms such as nonspecific pesticides, particulate matter, and tobacco smoke pollution and their related outcomes, there remained 99 AHS articles describing correlations between 62 specific chemicals and 46 diseases. We present a global view of the chemical–disease interaction landscape from AHS articles in Figure 4, highlighting significant positive and predictive correlations, significant negative correlations, areas of congruous and conflicting results, and perspective on gaps in conclusive information. Cancer was the most frequently reported disease category, with the highest number of correlations for prostatic neoplasms. Other broad disease categories frequently correlated with exposures included respiratory tract diseases and nervous system diseases.

**Figure 5:**
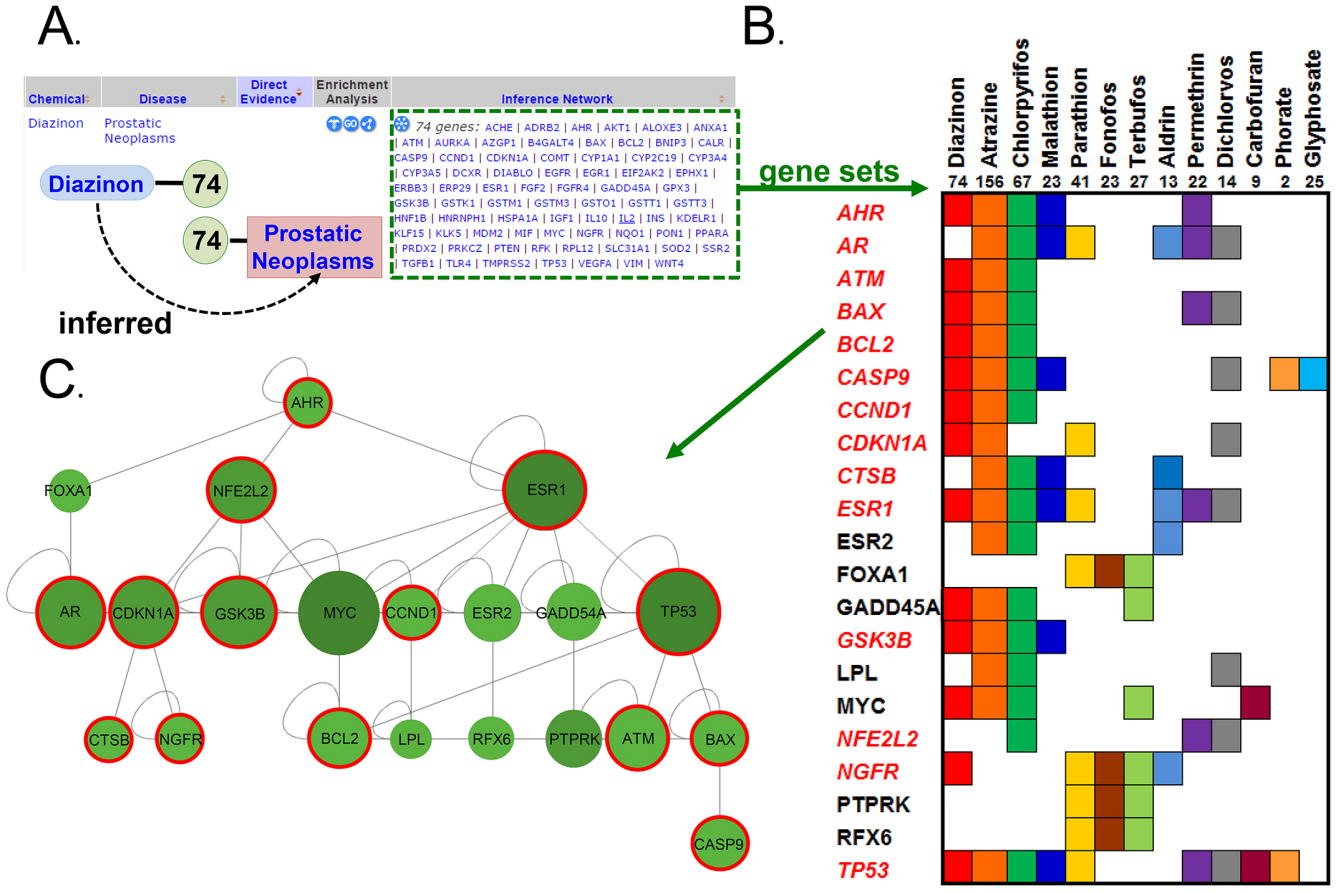
The core Comparative Toxicogenomics Database (CTD) informs exposure science by generating pesticide–prostate cancer interaction networks. (*A*) In the core CTD, the pesticide diazinon (blue oval) interacts with 74 genes (green circle) that also independently have an association with prostatic neoplasms (pink box), creating an inference network of 74 genes (green dotted box). (*B*) The number and gene set of each inference network for each individual pesticide linking it to prostatic neoplasms were collected (top row beneath pesticide name). In total, 21 genes (distributed over 13 pesticides) both interacted with 3 or more pesticides (colored boxes) and formed a non–self-interacting gene-gene network (*C*), providing a putative molecular subsystem for linking pesticide exposure to prostate cancer. Genes shown in red italics (*B*) and circled in (*C*) are also enriched in neurological disorders.

Integration of exposure data with core chemical–gene–disease information in the CTD provides new opportunities to explore potential mechanisms underlying exposures and health outcomes and to compare disease outcomes between exposure model systems and population-based studies. Core CTD data consist of a triad of chemical–gene, chemical–disease, and gene–disease relationships across diverse species. These data are integrated to construct chemical–gene–disease networks based on a common interacting set of genes, or “Inference Network,” which provides users with a possible underlying mechanism for the relationship (Figure 5A).

Among curated AHS data, 18 pesticides were associated with prostatic neoplasms; correlations were significant for 12 (aldrin, butylate, carbofuran, coumaphos, dichlorvos, fonofos, malathion, methyl bromide, parathion, permethrin, phorate, and terbufos) and hypothetical/predictive for 6 [atrazine, chlorpyrifos, diazinon, *S*-ethyl dipropyl(thiocarbamate) (EPTC), glyphosate, and metribuzin]. To augment the sparse mechanistic information provided in these studies, we leveraged the Inference Network genes that connected these pesticides to prostatic neoplasms in the core CTD. In total, 240 unique genes were associated with 16 pesticides (butylate and metribuzin did not have an Inference Network connecting them to the disease). To determine whether there was a common underlying molecular network, we restricted our analysis to genes that interacted with ≥ 3 of the pesticides. The resulting subset of 21 genes formed a common gene–gene interaction network (Figure 5C). Interestingly, 14 of these genes (Figure 5B) were also associated with neurological disorders (based on a disease enrichment query using the CTD’s Set Analyzer tool), supporting the positive correlations for several pesticides (e.g., butylate, phorate, and methyl bromide) with both prostatic neoplasms and Parkinson disease in the AHS data.

**Figure 6:**
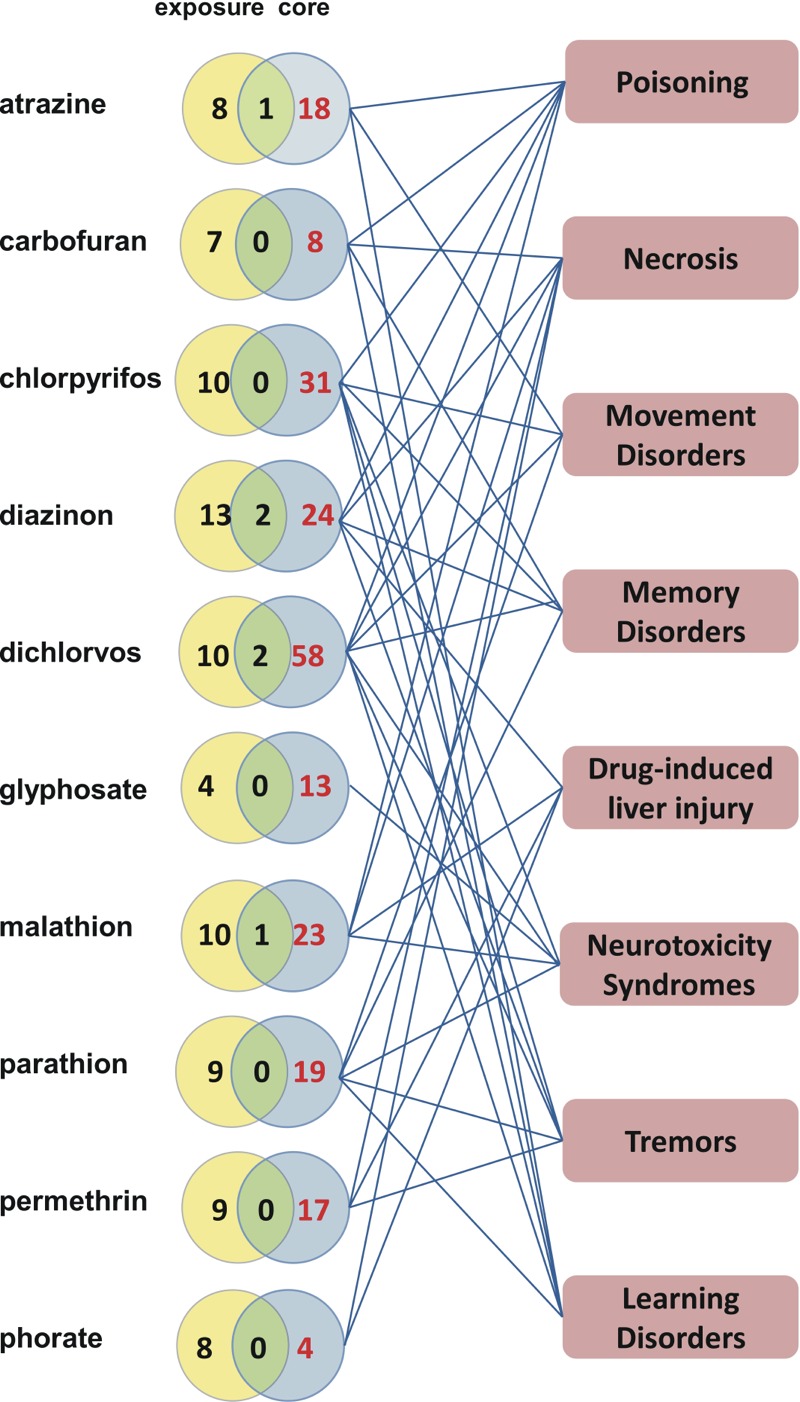
The core Comparative Genomics Database (CTD) potentially informs additional exposure science outcomes. Venn analyses comparing the number of diseases curated from exposure articles (yellow circles) to the number of diseases already curated in core CTD (blue circles) for 10 Agricultural Health Study (AHS) pesticides. Eight diseases from the core CTD (pink boxes) are shown connected to their associated pesticides. The additional diseases found in the core (red numbers) could putatively inform future exposure studies and help prioritize future research.

We compared disease outcomes for the 18 pesticides associated with prostatic neoplasms in AHS studies with core CTD data, which included model system studies. Ten of the 18 pesticides were associated with 130 additional diseases in the core CTD; 27% (35 of 130) were categorized as nervous system diseases or mental disorders, including conditions such as movement disorders, memory disorders, neurotoxicity syndromes, tremors, and learning disorders (Figure 6). These analyses could provide novel insights into cooccurring disease outcomes and their underlying mechanisms through integration of population-based and experimental data sets. Further, analysis of stressor–disease relationships in model systems could inform study design in humans.

## Discussion

Our new paradigm to manually curate and incorporate exposure science data into the CTD has many advantages, including *a*) responding to the need of the scientific community for centralization and harmonization of this critical information; *b*) providing new data perspectives that are not possible without integration (establishing novel connections between environmental chemicals, genes, diseases, biological processes, genetic networks, and pathways); *c*) facilitating meta-analyses of exposure data and providing opportunities to inform study design by allowing comparisons among experimental parameters such as detection methods, receptor attributes, and analytical approaches; *d*) enabling exposure data to leverage the CTD’s visual and analytical tools to explore relationships and elucidate potential connections between disparate data; *e*) adding real-world context to existing CTD information, providing instances of human exposures and disease outcomes to complement laboratory-based studies already curated in the core CTD; and *f*) most importantly, providing a free, user-friendly portal to explore exposure data within the recognized and well-established CTD framework. Cumulatively, this work supports the roadmap developed by the NRC to complement toxicology and risk assessment by “improving understanding of the link between environmental stressors and disease” (NRC 2012).

In this initial release, we have provided access to key components of our curated exposure data (stressor, receptor, country, medium assayed, marker assayed, assay measurements, outcome, and study summary). Access to the full spectrum of curated exposure data (see Table S1) will be available in an upcoming release and will enable more complex searching and comparison of exposure data across metrics such as geographical location or life stage. Centralization of these data will provide a more complete picture of environmental exposures, help identify gaps in our knowledge, and help refine or prioritize future studies. Together, these factors will contribute to a robust, literature-based exposome knowledge base that will continue to expand with our ongoing curation efforts.

Here, we demonstrated two ways that the CTD can be used to analyze integrated exposure data. First, AHS pesticides that are positively correlated with prostatic neoplasms were analyzed for gene networks previously curated for the core CTD. We discovered a submolecular interaction network that could potentially connect pesticide exposure to the disease and highlighted 21 genes that could be further investigated for *a*) differential expression in susceptible cohorts, *b*) polymorphisms that may predispose individuals to disease outcomes, or *c*) exposure-related epigenetic modifications that may influence exposure outcomes. Researchers could also explore enriched pathways or GO functional annotations among these genes using CTD tools. Second, we showed how the core CTD provided insights into additional diseases that may be associated with pesticides evaluated in the AHS. Susceptibility genes for these pesticides can be obtained using CTD search queries, and hypothetical mechanisms can be investigated in gene knockouts or other model systems. As curation expands, the potential for corroboration and bidirectional feedback between experimental and exposure studies will only increase.

An often-overlooked requirement of data integration projects is the need for semantic standards that enable consistent data representation. A recent NIEHS workshop (“Workshop for the Development of a Framework for Environmental Health Science Language,” http://www.niehs.nih.gov/about/visiting/events/pastmtg/2014/language/index.cfm) highlighted the lack of standards for environmental health science data and consequences for data integration and analysis (Mattingly et al. 2016). Major gaps exist, available standards can be redundant or are used inconsistently, and significant variability in study design and reporting methods challenge cross-study comparisons. We encountered each of these issues during our curation test phase, and attempts were made to identify semantic standards to capture exposure data in a consistent manner. Just a few examples of the data-related challenges encountered include, but are not limited to, extreme diversity in overall study objectives (ranging from epidemiological to measurements of compounds in house dust, etc.); dose measurements (described as distance from an exposure source, time exposed, estimated consumption of contaminated food source, particles per hand-wipe, etc.); biomarker measurements (reported as sums, averages, estimates, time-weighted, log-transformed, etc.); statistics (geometric means, arithmetic means, medians, percentiles, tertiles, etc.); tests for statistical significance (*p*-values, *p*-trends, odds ratios, incidence rate ratios, confidence intervals, etc.); smoking status (documented as cigarettes per day, pack-years, estimated time exposed to cigarette smoke, years since last cigarette, etc.); and even cohort age (described as mean, ranges, and often in imprecise terms such as “children,” “students,” “middle-aged,” “elderly”). Our policy is to report data as presented by the authors. Consequently, many data types might not be directly comparable in the absence of reanalysis by users. While the process of standardizing study attributes whenever possible is underway, we include a free-text field in our curation to capture additional important details. The diverse field of exposure data is an area that would benefit from community- and data-driven standardization efforts to ensure more widespread integration into exposome, toxicology, and risk-assessment research initiatives.

Although integration and centralization of exposure studies in the CTD provides an important first step in addressing existing gaps in epidemiological studies, limitations remain. For many of the curated studies, route of exposure was not addressed, or in some cases, multiple routes of exposure were suggested but not conclusively determined. Given the variability of these data, this information was not included in our curation paradigm, but it remains a very interesting and high-priority attribute for future consideration. Other important data types including emission, transport, and fate of the stressor, as well as human activities that define the timing, magnitude, and duration of contact with environmental media are beyond the scope of this project at the present time, but they remain important issues for future consideration. Likewise, biological, biomechanical, physical and psychosocial agents should be investigated as exposure stressors and in terms of how they modify susceptibility to other stressors. At the present time, the influence of genetic variants on an organism’s susceptibility to a stressor or exposure route is noted, but in many cases, the causality remains to be determined. In addition, we acknowledge that sample collection, measurement methods, limits of detection, accuracy, and quality of exposure data vary among studies. Although all of the studies are curated from peer-reviewed literature, we do not rank the quality of the data; however, we do provide links to the primary sources so that users can assess the relative strength of the data directly.

Future directions for the CTD’s exposure module include increased querying capabilities for exposure studies and user-directed displays of exposure-specific data by filtering content according to users’ preferences. CTD biocurators collect additional information such as receptor attributes (age, race, sex, and smoking status) that are not yet displayed on the Exposure Studies summary pages owing to space constraints, but this information will be included in future releases. These attributes will allow comparisons to be made among study populations and will promote new ways of combining data. Presently, geographic locations (i.e., country) are displayed on the Exposure Studies page for each curated article; these data will be expanded to include states and cities and will become a searchable field in a future release. In addition, future plans include color-coded geographic mapping of exposure data to promote region-specific analysis of stressors and outcomes.

## Conclusions

At the present time, the CTD includes curated exposure information integrated with data on chemicals, genes/proteins, diseases, biological processes, and molecular pathways to increase understanding of correlations between environmental exposures and human health, potential underlying mechanisms, and the exposome. The public release of a free, searchable, centralized database of exposure studies is an enormous first step in addressing gaps in exposure science access and analysis. We invite feedback from the public to maximize the functionality of our exposure module so that it can expand and evolve as an invaluable resource to the scientific community, providing critical insight into exposure stressor–receptor interactions, assessment of human health risks, and prioritization of toxicological research.

## Supplemental Material

(175 KB) PDF
