# Supplementary material for: Advancing Exposure Science through Chemical Data Curation and Integration in the Comparative Toxicogenomics Database
Source: Environ Health Perspect. 2016 May 12;124(10):1592–9. doi: 10.1289/EHP174 (PMC5047769; doi:10.1289/EHP174)
Supplement: (175 KB) PDF [file EHP174.s001.acco.pdf]

Environ Health Perspect
DOI: 10.1289/EHP174

Note to readers with disabilities: *EHP* strives to ensure that all journal content is accessible to all readers. However, some figures and Supplemental Material published in *EHP* articles may not conform to 508 standards due to the complexity of the information being presented. If you need assistance accessing journal content, please contact ehp508@niehs.nih.gov. Our staff will work with you to assess and meet your accessibility needs within 3 working days.

Supplemental Material

Advancing Exposure Science through Chemical Data Curation and Integration in the Comparative Toxicogenomics Database

Cynthia J. Grondin, Allan Peter Davis, Thomas C. Wiegers, Benjamin L. King, Jolene A. Wiegers, David M. Reif, Jane A. Hoppin and Carolyn J. Mattingly

Table of Contents

Part 1. Agricultural Health Study

Part 2. CTD Set Analyzer tool

Part 3. CTD MyVenn tool

Table S1. Key data fields and counts for CTD exposure curation

References

Part 1 Agricultural Health Study (AHS) (Alavanja et al. 1996). Following standard curation practices (Davis et al. 2011), interactions between chemical stressors and diseases in AHS papers were coded for type of relationship and significance in exposure statements. Each exposure statement describes a single interaction between a single stressor and a single outcome. When authors provided statistically significant evidence of a disease outcome as a result of a chemical exposure (author metrics varied, but often included odds ratios, relative risks, P-values and confidence intervals), this was curated as a positive or negative interaction between a stressor and disease outcome as appropriate. Interactions that showed a positive or negative trend that did not reach statistical significance (P-trend) were curated as hypothetical/predictive. Interactions that lacked correlation, as concluded by the authors, were coded as null interactions. In cases where the relationship between a stressor and outcome was evaluated but the results were inconclusive, the result was not recorded. All interactions between a single stressor and disease were sorted, counted, assigned a numerical value to represent the relationship, and visualized as a matrix; greater than two positive interactions were assigned a value of five, those with 1-2 positive interactions were assigned a value of two, single positive interactions combined with single negative interactions were assigned a value of 0.1, no interactions were assigned a value of 0, null interactions were assigned a value of -0.1, and negative interactions were assigned a value of -2. Because many stressor-disease combinations had multiple interactions, a single value was designated to best represent a consensus of curated literature. For heatmap analysis (Figure 4), hypothetical/predictive interactions were included with positive correlations. The R/ComplexHeatmap package was used for single linkage clustering of diseases by class, and then within class, by frequency.

Part 2 CTD *Set Analyzer* tool. To examine how integration of exposure data with CTD can add biological context to exposure information, we examined the set of 21 genes (Figure 5B) that interacted with 3 or more of the 16 AHS pesticides that have an association with Prostatic Neoplasms, and together form a gene-gene interaction network. Using CTD's SetAnalyzer tool, (http://ctdbase.org/tools/analyzer.go?q), 'Genes' were selected as the input type in part 1, the list of 21 genes were entered as the data set in part 2, 'Enriched Diseases' were selected as the type of analysis in part 3, and a threshold of 0.01 was entered as the corrected p-value in part 4.

Part 3 CTD *MyVenn* tool. CTD's *MyVenn* tool was used to examine how CTD core and exposure data sets complement each other and inform additional analyses for 18 AHS pesticides (aldrin, atrazine, butylate, carbofuran, chlorpyrifos, coumaphos, diazinon, dichlorvos, EPTC, fonofos, glyphosate, malathion, methyl bromide, metribuzin, parathion, permethrin, phorate, and terbufos). The complete CTD exposure data file was downloaded (http://ctdbase.org/downloads/#exposureevents) and sorted to identify diseases associated with each pesticide exposure and whether the relationship was a "positive correlation" or "prediction/hypothesis". Similarly, diseases associated with each of the 18 pesticides from core CTD data were downloaded and filtered to include only "M-type" (marker/mechanism) relationships. Separate analyses were conducted for each of the 18 pesticides, selecting 'Diseases' as the input type in part 1 of the *MyVenn* tool (http://ctdbase.org/tools/myVenn.go), and the respective disease sets generated from exposure and core curation entered in part 2 as Set 1 and Set 2, respectively. Results are described in Figure 6 for 10 of the 18 pesticides that showed differences between the two data sets.

Environ Health Perspect
DOI: 10.1289/EHP174

Table S1. Key data fields and counts for CTD exposure curation

Exposure Category	Curation Field	Vocabulary[a]	Validation Process	Curated data type	Counts[b]
**1. Curation Identifiers**	PubMed identification number	CV	pubMed ID, valid record must be processed for reference to be saved to database	Articles reviewed	1,712
	Curatable?	CV	must be yes/no	Articles curated	**1,067**
	Study title	FT/SD		Study titles	216
	Study years	CV	numerical value, collection start year must be less than or equal to collection end year		44,526
	Author email	FT	field not blank; format conforms to apache common email validation function		1,037
	Influencing health factors	CV	values match CTD controlled vocabulary	Influencing factors	11
	Author summary	FT		Author statements	**1,067**
**2. Stressor**	Chemical stressor	CV	must be a valid CTD vocabulary chemical name	Chemical stressors	**609**[c]
	Stressor source	CV	values match CTD controlled vocabulary	Stressor categories	6
	Stressor details	FT			20,269
	Stressor notes	FT			7,482
**3. Receptor**	Number of receptors	CV	must be numeric		40,404
	Receptor	CV	values match CTD controlled vocabulary	Receptor types	**12**
	Smoking status	CV	values match CTD controlled vocabulary	Smoking status types	4
	Age	CV	must be single numeric value or valid range format		34,202
	Gender	CV	values match CTD controlled vocabulary	Genders	2
	Race/ethnicity	CV	values match CTD controlled vocabulary of PhenX terms	Race categories	27
	Receptor notes	FT			20,609

	Methods	FT/SD		Methods	477
	Limit of detection	CV	if present, must be single numeric value or valid range format		18,319
	Detection frequency	CV	numeric value, must be < or = 100%		17,106
	Medium assayed	FT/SD		Media types assayed	**154**
	Chemical or gene marker	CV	must be a valid CTD vocabulary chemical or gene name	Chemicals/genes	**621[c] and 71**
	Exposure marker measurement level	CV	if present, must contain valid single numeric value, range, equal <LOD or equal <value1-value2 and contain LOD value and measurement units in respective fields	Exposure marker measurements	**51,982**
**4. Event**	Measurement units	FT/SD	must be present if marker measurement value is present	Measurement units	**285**
	Measurement statistical category	FT/SD		Statistical categories	**370**
	Assay notes	FT			21,450
	Country	CV	values match CTD controlled vocabulary from ISO 3166 table	Countries reported	98
	State (US) or province	CV	values match US state abbreviations	US states reported	**49**
	Geographic details	FT			28,896
	Event notes	FT			5,107
	Outcome correlation	CV	values match CTD controlled vocabulary	Outcome statements	2,901
**5. Outcome**	Disease or phenotype	CV	must be a valid CTD vocabulary disease name	Diseases/phenotypes	**245 and 146**
	Anatomical site of phenotype	CV	must be a valid CTD vocabulary anatomy name	Anatomical terms	44
	Outcome notes	FT			524

[a]CV = controlled vocabulary; FT = free text; SD = standardizing

[b]as of August 2015; data in bold are currently available in download file

[c]Stressors denote chemicals used in an exposure event, while chemical markers include one or more metabolites of the chemical stressors

References

Alavanja MC, Sandler DP, McMaster SB, Zahm SH, McDonnell CJ, Lynch CF, et al. 1996. The agricultural health study. Environ Health Perspect 104:362-369.

Davis AP, Wiegers TC, Rosenstein MC, Murphy CG, Mattingly CJ. 2011. The curation paradigm and application tool used for manual curation of the scientific literature at the comparative toxicogenomics database. Database (Oxford) 2011:bar034.
